# Supplementary material for: Peptides Targeting the IF1–ATP Synthase Complex Modulate the Permeability Transition Pore in Cancer HeLa Cells
Source: Int J Mol Sci. 2024 Apr 25;25(9):4655. doi: 10.3390/ijms25094655 (PMC11083241; doi:10.3390/ijms25094655)
Supplement: Supplementary file 1 [file ijms-25-04655-s001.zip › ijms-2895205-supplementary.pdf]

Supplementary Materials

Peptides Targeting the IF1–ATP Synthase Complex Modulate the Permeability Transition Pore in Cancer HeLa Cells

Martina Grandi [1,†], Simone Fabbian [2,†], Giancarlo Solaini [1], Alessandra Baracca [1], Massimo Bellanda [2,3] and Valentina Giorgio [1,*]

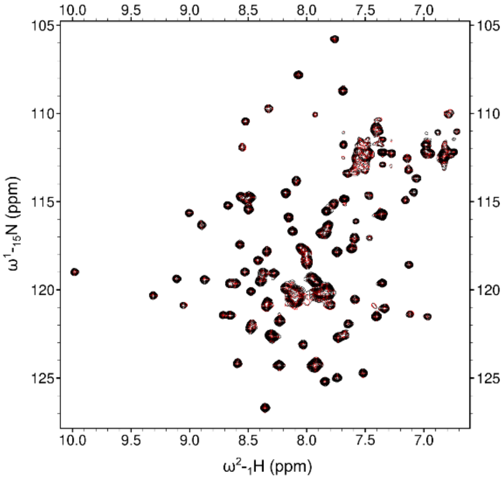

Figure S1. NMR spectra of the N-terminus of the OSCP subunit in the presence or absence of peptide IF1-O.3. ^{1}H-^{15}N SOFAST HMQC spectra of OSCP-NT (residues R6-G114) in the absence (red) and in presence (black) of a 10-fold molar excess of peptide IF1-O.3.

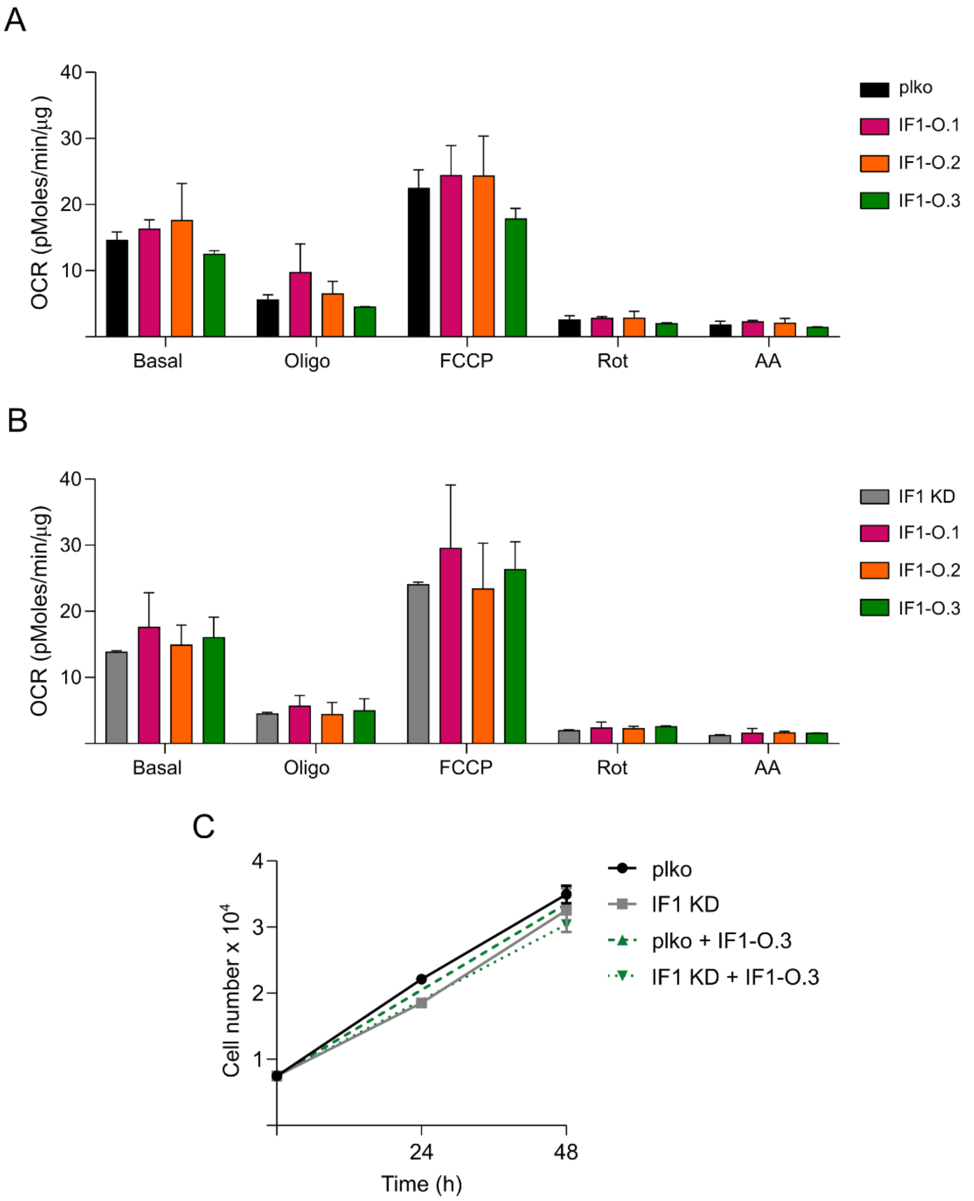

Figure S2. Effects of peptides IF1-O.1, IF1-O.2 and IF1-O.3 on mitochondrial respiration and cell proliferation. Normalized oxygen consumption rate (OCR) values per µg of protein of adherent plko (A) and IF1 KD (B) HeLa cells, treated with or without 30 µM of membrane-permeable peptides. OCR is measured before (Basal) and after treatment with oligomycin (Oligo), carbonyl cyanide p-(trifluoromethoxy) phenylhydrazone (FCCP), rotenone (Rot) and antimycin A (AA). In A and B, cells are incubated with TAT-IF1-O.1; TAT- IF1-O.2 and TAT- IF1-O.3 for 30 min before measurements. Data are mean ± SEM of three independent experiments. In C, cell growth is analyzed for 48 hours of plko and IF1 KD HeLa cells with or without 30 µM TAT- IF1-O.3. Data are mean ± SEM of three independent experiments.
